# Antiretroviral Treatment for HIV in Rural Uganda: Two-Year Treatment Outcomes of a Prospective Health Centre/Community-Based and Hospital-Based Cohort

**DOI:** 10.1371/journal.pone.0040902

**Published:** 2012-07-17

**Authors:** Walter Kipp, Joseph Konde-Lule, L. Duncan Saunders, Arif Alibhai, Stan Houston, Tom Rubaale, Ambikaipakan Senthilselvan, Joa Okech-Ojony

**Affiliations:** 1 Department of Public Health Sciences, School of Public Health, University of Alberta, Edmonton, Canada; 2 Department of Epidemiology and Biostatistics, School of Public Health, Makerere University, Kampala, Uganda; 3 Department of Medicine, Faculty of Medicine, University of Alberta, Edmonton, Canada; 4 Kabarole District Health Department, Fort Portal, Uganda; University of Cape Town, South Africa

## Abstract

**Background:**

In sub-Saharan Africa, a shortage of trained health professionals and limited geographical access to health facilities present major barriers to the expansion of antiretroviral therapy (ART). We tested the utility of a health centre (HC)/community-based approach in the provision of ART to persons living with HIV in a rural area in western Uganda.

**Methods:**

The HIV treatment outcomes of the HC/community-based ART program were evaluated and compared with those of an ART program at a best-practice regional hospital. The HC/community-based cohort comprised 185 treatment-naïve patients enrolled in 2006. The hospital cohort comprised of 200 patients enrolled in the same time period. The HC/community-based program involved weekly home visits to patients by community volunteers who were trained to deliver antiretroviral drugs to monitor and support adherence to treatment, and to identify and report adverse reactions and other clinical symptoms. Treatment supporters in the homes also had the responsibility to remind patients to take their drugs regularly. ART treatment outcomes were measured by HIV-1 RNA viral load (VL) after two years of treatment. Adherence was determined through weekly pill counts.

**Results:**

Successful ART treatment outcomes in the HC/community-based cohort were equivalent to those in the hospital-based cohort after two years of treatment in on-treatment analysis (VL≤400 copies/mL, 93.0% vs. 87.3%, *p = *0.12), and in intention-to-treat analysis (VL≤400 copies/mL, 64.9% and 62.0%, *p* = 0.560). In multivariate analysis patients in the HC/community-based cohort were more likely to have virologic suppression compared to hospital-based patients (adjusted OR = 2.47, 95% CI 1.01–6.04).

**Conclusion:**

Acceptable rates of virologic suppression were achieved using existing rural clinic and community resources in a HC/community-based ART program run by clinical officers and supported by lay volunteers and treatment supporters. The results were equivalent to those of a hospital-based ART program run primarily by doctors.

## Introduction

In sub-Saharan Africa, HIV patients receive antiretroviral treatment (ART) with combination antiretroviral drugs (ARVs) mainly through urban-based programs. Economic and geographic constraints severely limit access to hospitals for poor patients living in rural areas. Since much of the population of sub-Saharan Africa is rural, these factors lead to large inequities in the provision of ART services and makes universal access to ART difficult to achieve. Attempts to expand ART services to rural areas, including those in Uganda, have been constrained by the shortage of trained health professionals in these regions. Alternative approaches are required, including those that can engage and make use of rural community resources [1], [2]. Provision of ART services in Uganda has improved over time; however, major gaps in access remain, including in western Uganda [3].

Information on successful community-based ART programs in sub-Saharan Africa is limited. In Uganda, published results from other studies which used home-based or community-based care models show that a high number of HIV patients achieved suppressed HIV-1 RNA viral loads (VL) in these types of programs [4]–[6]. In one study, local citizens monitored treatment progress and adherence to medication in HIV patients receiving ART in Kampala, the major urban centre of Uganda [6]. The other studies were in rural areas but involved treatment models that required substantial external inputs (such as the provision of motorcycles for transport), which would limit the applicability and sustainability of ART provision in poor regions. There were no studies found that looked at community-based ART programs in rural areas that used locally available lower-cost resources. If community members could be safely involved in the provision of high quality ART care in rural areas in Uganda, where there are very few physicians and clinical officers are in short supply, then expanding ART services would be feasible as capable community members are generally very willing to participate in such programs and clinical officers can be relieved of routine follow-up by shifting this task to community volunteers.

We tested the utility and effectiveness of a HC/community-based model for delivering ART in a sustainable manner using local health centre and community support and resources by providing ART to a rural population in Rwimi subcounty, Kabarole District. We compared virologic outcomes with results of a well-established hospital-based ART program offered at the best practice regional hospital in Fort Portal, the district capital. The follow-up time for patients in both care models was two years.

We previously published the preliminary six-month treatment results from this project [7]. In this study we report the treatment results after two years of program operation, which is a more informative period of observation for the evaluation of a life-long treatment program.

### Objectives of the Study

The specific objectives of the study were as follows:

**Objective #1:** To assess the effectiveness of a rural HC/community-based ART program in Rwimi subcounty, Kabarole district; and

**Objective #2:** To compare the treatment outcome (VL) in the rural HC/community-based ART program with a well-established hospital-based ART program offered at a best practice regional hospital in the district capital.

We tested the hypothesis that a HC/community-based ART program in a rural subcounty can provide a high standard of care and can produce outcomes equivalent to a physician-centered ART model delivered in an urban hospital setting.

## Methods

### Study Design

This was a comparative cohort study where participants were followed-up according to where they chose to receive health care services. The study group consisted of an inception cohort of treatment-naïve HIV patients initiating HC/community-based ART in the study area. The comparison group consisted of an inception cohort of treatment-naïve HIV patients starting ART in a regional hospital. The first line treatment provided by the Ugandan national HIV/AIDS program consisted of stavudine, lamivudine, and nevirapine (or efavirenz for patients on rifampicin) to be taken twice daily. All patients were also prescribed daily co-trimoxazole. Each patient was followed up for two years from the start of treatment.

### Background Information of the Study Area

An estimated 22,400 persons living with HIV (PLWHIV) reside in Kabarole district in western Uganda [8]. HIV prevalence in the district is 11.6% which is above the national average of 6.4% in adults [8], [9]. Rwimi subcounty (population 25,000) is located in the southernmost corner of Kabarole district, 50 km away from the nearest hospital offering ART. The main sources of income are subsistence farming and trading. Very few PLWHIV in Rwimi subcounty were on ART before our project started; those who were on treatment obtained it from the hospital in Fort Portal. A Health Centre III in Rwimi subcounty was selected as our pilot site. The selected health centre runs a general outpatient clinic for patients with any medical problems, including HIV infections, within the catchment area of the clinic. The health centre is staffed with two clinical officers, two nurses and one midwife and sees on average 50–70 outpatients per day. The health care workers were trained in ART as part of the pilot project. The health centre was accredited for ART provision by the Ugandan government with the help of the research team (this was the first Health Centre III in Uganda to receive this designation).

### Study Population and Sample Size

Consecutive treatment-naïve PLWHIV with a CD4 cell count of 200/µL or less and/or clinical symptoms were enrolled in both cohorts as they presented themselves at the two study sites. There were no refusals or exclusions among those who fulfilled the inclusion criteria. The intended sample size was approximately 200 patients in each cohort. Patients attending the health centre at Rwimi were eligible for enrolment if they fulfilled the following criteria: resident in Rwimi subcounty, age of 18 years and older, treatment-naïve, eligible for ART according to the Uganda national HIV guidelines, willingness to accept daily treatment support by family/friends and willingness to be visited by a trained community volunteer once a week. Participants were identified through the clinic when they presented with symptomatic HIV infection, through antenatal care, or through voluntary counselling and testing (VCT) which was expanded during the study period. A Ugandan physician trained and experienced in ART oversaw the enrollment of patients into the study to ensure that the clinical officers and community volunteers delivered ART care according to the national guidelines standard. The schedule for a regular follow-up visit at the health centre for community-based patients was set at every six months, at which time they were seen by a clinical officer, and necessary laboratory investigations were performed.

For the hospital-based cohort recruitment criteria were: being a resident of Kabarole district, age of 18 years or older, treatment-naïve, and eligible for ART according to the Ugandan national HIV guidelines. Patients in the hospital-based cohort received the same care delivered to all other HIV patients in the hospital program except for viral load measurements at 6 and 24 months. Regular follow-up visits for hospital-based patients were scheduled every month, when they were seen by a physician or by a clinical officer when a physician was not available. Laboratory tests were conducted every six months. The hospital program’s own physicians enrolled patients into the study. The loss of follow-up in both groups was defined as death or the inability to contact a patient after missing an appointment.

Enrollment in the HC/community-based cohort started in March 2006 while enrollment for the hospital-based cohort started in April 2006. By November 2006 the hospital-based cohort was fully recruited (n = 200) and by May 2007 185 patients had been recruited in the HC/community-based cohort. Because of slower than anticipated recruitment, we decided to cap recruitment at 185 in the HC/community-based cohort due to time and budget constraints.

Sample size calculations were made using the main variable of interest, namely treatment success/failure based on suppressed VL. In the HC/community-based cohort we expected the treatment success to be 80% as per literature review. Our intended sample size of 200 patients in each cohort allowed us, with 0.80 power and a significance level of 0.05, to detect a difference of ±5% in successfully treated patients between the HC/community-based cohort and the hospital-based cohort which we believed would be a meaningful and practical difference [10].

### Description of the Intervention

The intervention was designed to provide ART to rural, impoverished PLWHIV most of whom would otherwise have had no access to treatment due to high transport costs imposed by hospital-based treatment. Duff et al. have shown in a previous study from the same area that transport costs to the hospitals providing ART and other treatment associated costs were the main barrier to accessing ART [11]. Community members in Rwimi were asked if they would participate in this program as unpaid volunteers. Forty-one volunteers agreed to participate and were provided with training on ART benefits, risks and limitations, the critical importance of adherence to the medication and the expected adverse reactions to ARVs as well as how to monitor patient adherence using pill counts. The volunteers were asked to make weekly visits to their patients. Each volunteer had on average 4–5 patients in order to keep the workload manageable. During the weekly visits, the volunteers performed a pill count and assessed the presence of clinical problems and adverse reactions. Volunteers were asked to refer the patient with clinical problems and/or adverse reactions to ARVs to the clinical officer at the health centre. At these visits the volunteers recorded data on their findings on standardized forms. Patients with medical conditions which required specialized treatment not available in the health centre were referred by the clinical officer to the regional hospital. On a monthly basis the volunteers obtained ARVs from the health centre and delivered these to patients. In addition, they provided information on HIV/AIDS prevention to their patients and distributed condoms. When patients were recruited, they were also asked to identify a family member/friend as their treatment supporter to provide daily support for treatment adherence. Patients and their treatment supporters were counseled together on important aspects of treatment including lifelong duration of treatment, possible adverse reactions of the drugs and the need for high adherence to the medication. Treatment supporters were asked to remind patients to take their medications and record these on a patient log that was provided by the study. The volunteer logs were entered into a Microsoft Access database.

The motivation of the volunteers was based on the recognition and support they received from the health care program and the community. Basic supplies required for their work were provided, e.g. a bicycle, raincoats and gumboots. An annual volunteer appreciation day was organized with participation of the entire community and its leaders. The volunteers did not receive any cash payments. Monthly meetings of all volunteers were held with a volunteer administrator, where problems were discussed, solutions sought and where the report forms were delivered and checked by the administrator. The boots, raincoats and bicycles along with volunteer coordination, enrollment by a physician and support activities described above were the only external material inputs by the study, over and above the resources routinely available to this publicly funded clinic. The study also provided an emergency supply of ARVs and co-trimoxazole for stock outs.

The hospital clinic was a busy outpatient HIV clinic with an average of 80–100 patients per day, where two physicians handled the initiation and the monthly follow-up of ART patients. In case a physician was not available, a clinical officer dealt with the routine follow-up.

### Data Collection and Analysis

The primary outcome measure at 24 months after starting ART in each patient was virologic suppression (HIV-1 RNA viral load [VL] ≤400 copies/mL). A secondary outcome measure was the increase in CD4 cell count/µL between baseline and follow-up tests. CD4 cell count tests (FACScount, Becton, Dickinson and Company) and HIV-1 RNA viral load tests (Cobas Amplicor HIV-1 Monitor test, Roche Molecular Systems) for both cohorts were carried out in the laboratory of the Joint Clinical Research Centre (JCRC) in Fort Portal, which is part of a network of laboratories with international quality control (tests for HIV-1 RNA levels≤50 copies/mL were not available in this laboratory at the time). Demographic data were derived from the standardized Ministry of Health clinical data sheets completed during the initial patient examination. Adverse outcomes were extracted from clinical records kept in the clinic and in the hospital. Data were entered into Microsoft Excel spreadsheets and Microsoft Access database and checked for inconsistencies or exceptional values. Adherence to the medication schedule was measured based upon weekly pill counts, where the actual number of pills remaining were compared with the calculated expected pill count; both data were recorded in the volunteer adherence logs.

Statistical procedures included descriptive, univariate, and multivariate analyses. The statistical significance of the differences in the demographic, clinical, and immunological characteristics were tested using chi-squared tests for categorical variables, two sided-independent samples t-tests or Mann-Whitney U tests for continuous variables and survival analysis with the Kaplan-Meier curve and the estimation of the Hazard Ratio (HR). The association between virologic suppression and independent variables was determined using multiple logistic regression. The predictor variables considered for the analysis were those that were significant at *p = *0.2 in the univariate analysis or that were clinically important. The main interest in the multiple logistic regression analysis was to determine the association between virologic suppression and the dichotomous indicator of cohorts (HC/community-based or hospital-based). To test if both treatments were equivalent, the limits of equivalence were assumed to be ±15%. Based on this assumption, the two treatments could be claimed to be equivalent if the difference between the proportion of success (θ) of the treatment 1 and treatment 2 lies in the interval −15% (θ_L_) to +15% (θ_U_). We tested the two one-sided hypotheses H_0_: θ = θ_L_ against the alternative H_0_: θ>θ_L_ and H_0_: θ = θ_U_ against the alternative H_0_: θ<θ_U_, respectively. Equivalence between the two treatments was asserted if both hypotheses were rejected. Statistical analysis was performed using Stata (version 9.0) statistical software [12]. The significance level for all statistical tests was set at 0.05.

### Ethics Approval

Ethics approval was provided in Canada by the Health Research Ethics Board, University of Alberta, Edmonton, and in Uganda by the Uganda National Council of Science and Technology, Kampala, the School of Public Health, Makerere University, Kampala and the District Health Officer for Kabarole District. Each participant was informed about the study and provided written consent or a thumbprint. The volunteers and treatment supporters also provided written consent or thumbprint.

## Results

**Figure 1:**
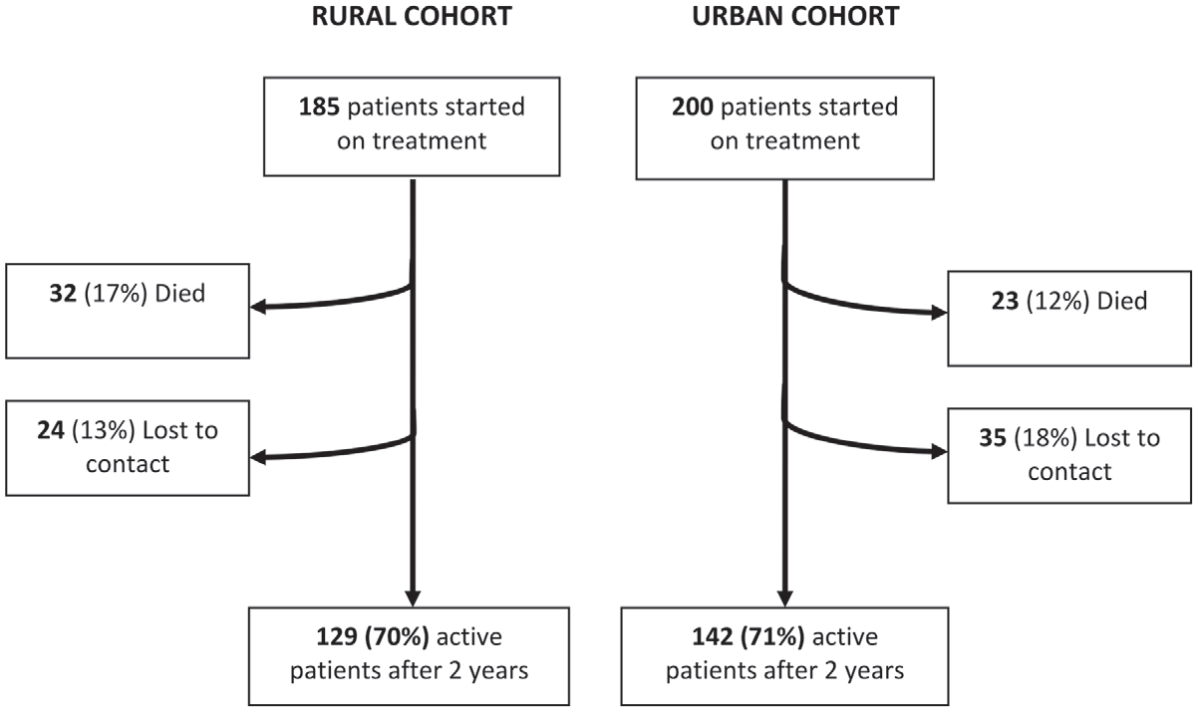
Follow-up of patients (died, lost to contact) in the community-based and hospital-based cohorts over the study period of two years.

Of the 185 patients who were recruited in the HC/community-based group, 24 (13%) were lost to contact and 32 (17%) died during the two-year follow-up (Figure 1). In the hospital-based group of 200 patients, 35 (18%) were lost to contact and 23 (12%) were known to have died during the same follow-up period.

Among the patients who remained in the study at two years, significant differences were observed in mean age (*p = *0.049), education (*p = *0.042), marital status (*p<*0.001) and occupation (*p<*0.001) between HC/community-based and hospital-based patients. As shown in Table 1 and 2, the baseline characteristics of those patients who were studied at two years and those who were lost to follow-up and/or died in the two groups were similar except for the initial median CD4 cell count, which was significantly lower in those patients who were lost in the HC/community-based group.

**Table 1:** Demographic, clinical and immunological baseline characteristics for HC/community-based and hospital-based patients.

	Baseline characteristics of patients				
	Community Based n = 185∞		Hospital Patients n = 200^Ω^		
Characteristic	*n*	(%)	*n*	(%)	*p* value^§^
Sex					
Male	76	(41.1)	87	(43.5)	0.631*
Female	109	(58.9)	113	(56.5)	
Education					
None	56	(30.6)	46	(23.2)	0.027*
Primary	105	(57.4)	109	(55.1)	
Secondary or higher	22	(12.0)	43	(21.7)	
Marital Status					
Single	26	(14.2)	60	(30.2)	<0.001*
Married	73	(38.9)	84	(42.2)	
Other	84	(45.9)	55	(27.6)	
Occupation					
No occupation	41	(22.4)	58	(29.4)	<0.001*
Farmer or non-professional	117	(63.9)	36	(18.3)	
Businessman or professional	25	(13.7)	103	(52.3)	
Age in Years, mean (SD)	36.8	(8.9)	34.8	(11.5)	0.147†
CD4 cell count (cells/mm^3^ blood), median (25^th^–75^th^ percentile)	137	(81–193)	131	(66–200)	0.579‡

SD = Standard Deviation.

* χ^2^ test.

† Two-tailed t-test.

‡ Mann-Whitney U Test.

∞ missing data on education, marital status and occupation for 2 patients ^α^ missing data on education for 2 patients, marital status for 1 patient and occupation for 3 patients.

**Table 2:** Demographic, clinical and immunological characteristics for HC/community-based and hospital-based patients who died and/or were lost for contact.

	Baseline characteristics of patients who died or were lost to follow-up,				
	Community Based *n = *56∞		Hospital Patients *n = *58^Ω^		
Characteristic	*n*	(%)	*n*	(%)	*p* value^§^
Sex					
Male	28	(50.0)	28	(48.3)	0.854*
Female	28	(50.0)	30	(51.7)	
Education					
None	19	(35.2)	16	(28.1)	0.527*
Primary	26	(48.1)	27	(47.4)	
Secondary or higher	9	(16.7)	14	(24.5)	
Marital Status					
Single	14	(25.9)	17	(29.8)	0.604*
Married	18	(33.3)	22	(38.6)	
Other	22	(40.8)	18	(31.6)	
Occupation					
No occupation	13	(24.1)	14	(24.6)	<0.001*
Farmer or non-professional	32	(59.3)	9	(15.8)	
Businessman or professional	9	(16.7)	34	(59.6)	
Age in Years, mean (SD)	34.8	(11.5)	35.1	(10.3)	0.877†
CD4 cell count (cells/mm^3^ blood), median (25^th^–75^th^ percentile)	125	(69–184)	171	(50–95)	0.216‡

SD = Standard Deviation.

* χ^2^ test.

† Two-tailed t-test.

‡ Mann-Whitney U Test.

∞ missing data on education, marital status and occupation for 2 patients ^α^ missing data on education, marital status and occupation for 1 patient.

**Figure 2:**
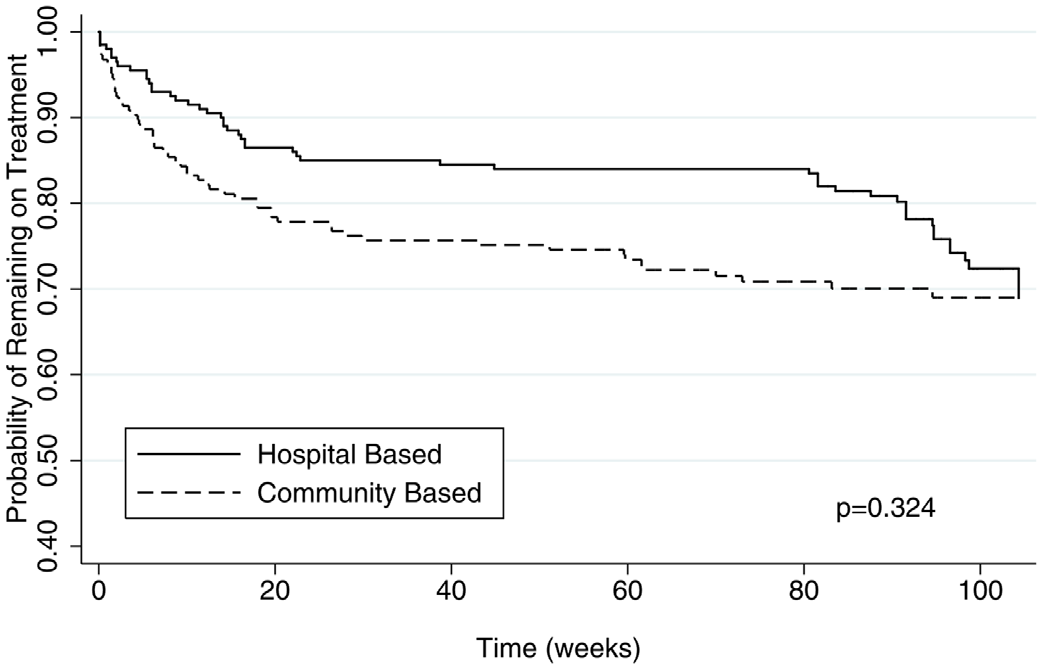
Kaplan-Meier curves for the patients who died and were lost to contact in the community-based and hospital-based cohorts over the study period of two years.

Crude mortality was higher in the HC/community-based cohort compared to the hospital-based cohort, though this difference was not statistically significant (17.3% vs. 11.5%, *p* = 0.10). The median time to death for hospital patients was 57 days (25^th^–75^th^ percentile = 6–102 days), and for community patients it was 63 days (25^th^–75^th^ percentile = 14–140 days). This difference was not statistically significant (p = 0.633). The unadjusted Hazard Ratio (HR) for death in the HC/community-based groups was 1.20 (95% CI = 0.83 to 1.73, p = 0.327). This comparison of mortality has its limitation, as the lost to follow-up group in both cohorts includes an unknown number of deaths. Figure 2 shows that the community-based cohort experienced an early loss or death of patients while the hospital cohort experienced more losses and deaths after approximately 80 weeks of treatment.

The median VL at baseline was 172,500 copies/ml in the HC/community-based cohort. Baseline VL tests were not available in the hospital-based cohort. The median baseline CD4 cell count of those who died was significantly lower compared to survivors in both cohorts combined (98 cells/mm^3^ vs. 143 cells/mm^3^, *p* = 0.040).

None of the patients experienced severe adverse reactions (assessed by a physician/clinical officer in the hospital-based group and by volunteers and a clinical officer in the HC/community-based group), that required a change to second-line treatment according to the prevailing standards of practice in Uganda and limited treatment alternatives. Seventeen patients in the HC/community-based cohort developed a skin rash (six patients in the hospital-based cohort) and 23 patients in the HC/community-based cohort presented with peripheral neuropathy (51 in the hospital-based cohort). The diagnosis of neuropathy is subjective, so misdiagnosis or over-diagnosis is possible. In the on-treatment analysis at the two year endpoint, 93.0% (120 out of 129 patients) in the HC/community-based cohort and 87.3% (124 out of 142 patients) in the hospital-based cohort respectively achieved virologic suppression (*p* = 0.12 in bivariate analysis).

When an intention-to-treat analysis was applied, which assumed that those who were lost to follow-up were not virologically suppressed, the corresponding numbers for virologic suppression at two years were 64.9% (120 out of 185 patients) in the HC/community-based cohort and 62.0% (124 out of 200 patients) in the hospital-based cohort (*p* = 0.673). The median increase in CD4 cell counts were 217 cells/µL after the two-year endpoint in HC/community-based patients and 193 cells/µL in hospital-based patients (*p* = 0.199). In the on-treatment analysis a multiple logistic regression analysis was carried out adjusted for age, sex, marital status, and baseline CD4 cell count levels (Table 3). The model showed that the only factor significantly associated with virologic suppression was being enrolled in the HC/community-based cohort; patients in the HC/community-based cohort were 2.47 times more likely to have a virologic suppression compared to patients to hospital-based patients (95% CI for OR = 1.02–6.04, *p = *0.046). Based on a 15% limit of equivalency, the treatments in the HC/community-based and hospital-based group were equivalent.

**Table 3:** Odds ratios (ORs) and 95% confidence intervals (CIs) from univariate and multivariate logistic regression for the association between the factors and with treatment success (VL ≤400 copies/mL) after 24 months.

	Univariate analysis			Multivariate analysis*		
Characteristic	OR	(95% CI)	*p* value	OR	(95% CI)	*p* value
Cohort						
Hospital-based	1.00			1.00		
Community-based	1.94	(0.84, 4.48)	0.12	2.47	(1.01, 6.04)	0.046
Age (years)	0.98	(0.94, 1.02)	0.35	0.99	(0.95, 1.04)	0.66
Sex						
Male	1.00			1.00		
Female	1.26	(0.56, 2.80)	0.58	1.54	(0.64, 3.75)	0.34
Marital status						
Single	1.00			1.00		
Married	0.84	(0.25, 2.80)	0.78	0.57	(0.15, 2.23)	0.42
Other	0.52	(0.16, 1.68)	0.27	0.28	(0.07, 1.13)	0.07
Education						
None	1.00			–	–	–
Primary	1.15	(0.47, 2.80)	0.76	–	–	–
Secondary or above	2.71	(0.55, 13.44)	0.22	–	–	–
Occupation						
No occupation	1.00					
Farmer or non-professional	0.84	(0.30, 2.37)	0.73	–	–	–
Businessman or professional	0.68	(0.24, 1.98)	0.48	–	–	–
Baseline CD4 cell count†	1.00	(0.99, 1.00)	0.22	1.00	(0.99, 1.00)	0.35

* Only those characteristics that were clinically important or significant at *p* = 0.2 in the univariate analysis were subjected to multivariate analysis.

† For 10 units increase in CD4 cell count.

Adherence was analyzed only in the HC/community-based cohort, as adherence information was not consistently recorded in the charts of the hospital patients. HC/community-based patients had excellent overall adherence rates, which were maintained at over 95% throughout the study period.

Our assessment of the volunteer program showed that 39 out of 41 volunteers remained active in the program over the entire study period. The volunteers made on average 90% of their planned home visits and spent on average 20 minutes in the home of the patients per visit. Average travel time to the homes of all patients was 88 minutes (SD = 38 min, range = 30–260 min). Monthly management meetings between the volunteers and the volunteer administrator took place regularly as planned with an attendance rate no lower than 85% at each meeting. Unscheduled visits of patients in the HC/community-based cohort were as follows: sixteen patients (six with HIV-related medical conditions) were referred to the regional hospital, while 38 patients were referred to the health centre by the community volunteers or decided to attend the health centre on their own without referral. The number of unscheduled visits of hospital patients to the HIV clinic in the hospital was not systematically recorded.

## Discussion

Our study results show that in rural western Uganda, ART can be delivered through a HC/community-based program using existing resources with treatment outcomes equivalent or marginally better than to those of the best-practice hospital in the district. This comparison hospital was part of a nationwide program through the Joint Clinical Research Centre (JCRC) and therefore represents high national standards of ART provision in Uganda. Our study findings demonstrate that a HC/community-based HIV treatment program can be a feasible, safe and effective option for increasing access to ART in rural areas. The positive results in our HC/community-based cohort are best explained as resulting from the support provided to the patients by volunteers, treatment supporters and the community at large. These support mechanisms were not available in the hospital ART program, as support for adherence in the hospital program was limited to the contacts during the monthly hospital visits. The advantage of the HC/community-based model is that it not only delivered equally successful treatment outcomes but also delivered treatment to rural patients who may have otherwise not been able to access the hospital-based treatment program because of distance and travel associated costs [11] and required much less use of scarcer physician time.

The proportion of virologic suppression in our HC/community-based cohort of 93% on active on-treatment patients and 68% in intention-to-treat patients, compare favorably with results from several other studies conducted in Uganda. Our results are superior to Chang et al. who reported 86% virologic suppression in active clients and 59% virologic suppression in intention-to-treat analysis after two years from a home-based ART program in a city program in Kampala, the nation’s capital [6]. At Mulago Hospital, Kampala, Kamya et al. followed up 454 HIV patients on ART. After one year of treatment 86% of active clients showed virologic suppression and in intention-to-treat-analysis this number was 75% [13]. Treatment results from the Jinja-based trial reported by Jaffar et al. are better than ours, showing 76% of patients with virologic suppression in intention-to treat analysis after 42 months of observation time [5]. The DART Virology Group and Trial Team found a virologic suppression rate of 74% in African adult patients from Uganda and Zimbabwe after 24 weeks [14]. All the Ugandan studies used the same threshold of VL≤400 copies/mL and comparable treatment schedules. One community-based study from South Africa had a very similar treatment outcome in intention-to treat-analysis to our study with 69.7% of patients having a suppressed VL [15]. In South America, virologic suppression after one year was observed in 76.7% of HIV patients in a community-based urban ART program in the health district of Lima, Peru [16]. Comparing ART program results from developed countries, the Swiss HIV Cohort study with an enrolment of 2,647 HIV patients revealed a virologic suppression rate of 79.1% in patients after two years. In contrast to our ART regimen, they used a triple combination with a protease inhibitor. In addition, 52% of those patients changed treatment at least once [17]. Overall, the results from our HC/community-based study fit well within the range of results from these studies in varied settings.

Important to our study is the fact that it was not conducted in a controlled research setting as were most other studies, but rather based in a typical rural Ugandan area which was supported by a publicly funded clinic where all HIV patients who presented themselves consecutively were treated according to the Uganda’s national HIV guidelines without any pre-selection. Rosen et al., in their assessment of ART program outcomes, distinctly differentiate between research ART programs and non-research ART programs and state that treatment outcomes in the former are generally better as a result of patient selection, enhanced resources and optimal project conditions and that the results of such programs therefore cannot be readily extrapolated to general health services [18]. The treatment results from our project which was integrated into a rural health service setting should be interpreted with consideration for this realistic context. Furthermore, engaging lay volunteers in our study for routine ART support activities minimized additional workload for the existing clinic staff, thus allowing them more time to attend to other patients with non-HIV/AIDS related health issues.

We could not definitively determine the mortality after two years in both cohorts as we were unable to follow-up all patients who were lost to contact to determine if they died. Therefore, we could not conduct a definitive analysis of mortality. If we use the result of 17% of mortality in the HC/community-based cohort at the two years endpoint, it compares well with the study by Chang et al. where mortality was 18% [6]. In comparison with other facility-based studies, the two year mortality of ART patients was 13.2% in Malawi in 2008 [19], 13.7% in South Africa [15], and 21% in a Ugandan fee-for-service program [20]. The higher mortality in our HC/community-based patients compared to our hospital patients could be explained by their lower educational level (see Table 1), as well geographical barriers to accessing acute care for opportunistic infections or other acute illnesses (such as TB) particularly in the vulnerable early period of ART provision. The differences in mortality could also be attributed to differences in ascertainment, e.g. the inability of the hospital program to properly ascertain mortality in their cohort compared to community volunteers doing home visits.

The difference between our study and the Jaffar et al. study [5] was that their study was implemented within the Ugandan Support Organization (TASO), which is better resourced than the Ugandan Government health care services and where qualified field officers (with formal post-secondary education) with motorcycles and four weeks of training visited HIV patients at home. We used local community volunteers who were provided with a two-day training program, follow-up training during the monthly meetings, and bicycles. The Chang et al. study [6] was implemented in an urban setting, where conditions are usually more favorable for the implementation of health services than in disadvantaged rural regions. The interpretation of their study results and ours should take these differences into account.

In their comprehensive review of community-based ART programs, Wringe et al. identified several factors which they found to be important predictors for successful community-based ART provision: program retention of volunteers, adequate support for volunteers, a functioning referral service, community acceptance and appropriate program costs [21]. Using these factors, our project results measure up well, as retention of volunteers was high, adequate material, technical and moral support was provided to the volunteers, and the referral of patients was a function of integration with the government health care service. In addition there was a wide-spread acceptance of our program by the communities in the catchment area of the health centre with no resistance to the program reported.

### Limitations

First, we did not have a randomized study design because randomization of patients was not possible. Rural patients would have been unable to access care at the distant regional hospital and patients who were close to the hospital would not have been willing to access rural services. Second, loss of follow-up may have affected the interpretation of the study. When we examined the baseline demographic and immunological characteristics of the patients lost to follow-up in both cohorts, we found they were not significantly different from those of the remaining participants. This may indicate that a possible selection bias was probably similar in both cohorts and therefore did not likely compromise the comparability of results for those remaining on treatment in both cohorts. Finally, information on adherence to ART and adverse reactions to antiretroviral drugs was not available in the hospital-based cohort. Therefore, adherence could not be compared with the hospital-based cohort.

### Conclusions

Our study shows that a non-physician-centered, non-hospital-based care model can deliver ART with the required quality of service, acceptable safety of patients, and with the treatment outcomes equivalent to those found in the facility-based ART program in the study area hospital. In the HC/community-based program the physician saw patients only once, unless referral was required for a clinical complication. With experience we didn’t think that the physician was necessary and in the post-study operation of Rwimi health centre, patients were started on ART by the clinical officer alone.

Our care model made ART more easily accessible to patients for whom distance and transportation costs would otherwise have been major barriers to hospital-based care [11]. Our model favors the replicability of ART services in rural areas by shifting ART program routine activities from higher trained to lower trained health staff and to community volunteers (for example, a hospital patient had on average 24 routine visits to a physician/clinical officer while a HC/community-based patient had an average of four routine visits to a clinical officer within the duration of the study). Lower trained cadres of health staff and community volunteers are more likely to be available in rural areas. Consequently our model is easier to implement in these areas. Our program prototype, with its health centre/community-based approach and modest study inputs could facilitate its replication more generally, thus enabling the scale-up of HIV treatment programs in rural health care settings of Uganda and elsewhere in sub-Saharan Africa.
